# Psychopathological profile before and after bariatric surgery

**DOI:** 10.1038/s41598-023-43170-2

**Published:** 2023-09-27

**Authors:** Tura Benítez, Assumpta Caixàs, Pere Rebasa, Alexis Luna, Sara Crivillés, Teresa Gutiérrez, Joan Deus

**Affiliations:** 1 Institut d’Investigació i Innovació Parc Taulí (I3PT-CERCA), Parc del Taulí, 1, 08208 Sabadell, Barcelona, Spain; 2 Mental Health Department, Hospital Universitari Parc Taulí, 08208 Sabadell, Spain; 3 Endocrinology and Nutrition Department, Hospital Universitari Parc Taulí, 08208 Sabadell, Spain; 4 Department of Medicine, Universitat Autònoma de Barcelona, 08208 Sabadell, Spain; 5 Department of Surgery, Hospital Universitari Parc Taulí, 08208 Sabadell, Spain; 6 Department of Clinical and Health Psychology, Universitat Autònoma de Barcelona, 08193 Bellaterra, Spain; 7 MRI Research Unit, Department of Radiology, Hospital del Mar, 08003 Barcelona, Spain

**Keywords:** Psychology, Endocrinology

## Abstract

Presurgical psychopathological assessment usually focuses on detecting severe mental disorders. However, mild intensity psychopathology and eating behaviour pattern may also influence postsurgical outcomes. The aim was to identify psychopathology and eating behaviour pattern in candidates prepared for bariatric surgery compared to a normative population before and after surgery. A cohort of 32 patients seeking bariatric surgery in a university hospital between March 2016 and March 2017 were evaluated with Personality Assessment Inventory (PAI), 36‐item EDE‐Q and BES before and after surgery. Thirty-two patients before and 26 one year after surgery were included. The PAI presurgical psychometric profile suggested a mild mixed adjustment disorder focused on somatic complaints. After surgery, patients improved in somatic complaints (*p* < 0.001), and depression (*p* = 0.04). Related eating disorders were more common than those of the normative group and improved significantly after surgery in scores for compulsive intake (BES *p* < 0.001) and overall key behaviours of eating disorders and related cognitive symptoms (EDE-Q/G *p* < 0.001). In our cohort ready for bariatric surgery a mild psychopathological profile is still present and becomes closer to that of the normative group after surgery. Further studies are needed to evaluate the effects of mild psychopathology on outcomes after bariatric surgery.

## Introduction

Bariatric surgery (BS) is an effective treatment for obesity that is being used increasingly worldwide; however, physical changes before or after surgery can induce significant stress^1^. People with severe obesity awaiting BS may refer psychiatric symptomatology^2^ and poor quality of life^3^. Moreover, personality traits and different degrees of psychopathology may influence outcomes after BS^1^. Nevertheless, research regarding psychopathology and psychological factors for prediction of successful weight loss have been inconsistent so far. In this sense, some studies^4–7^ but not all^8,9^ have reported a negative relationship between presurgical psychopathological symptoms, especially depressive and anxiety disorders with postsurgical weight loss.

Also, BS has been shown to ameliorate health-related quality of life and eating disorder symptoms. However, the correlation of these changes with weight loss is not uniform, suggesting that additional factors have an impact on postoperative outcomes^10^. A growing body of evidence indicates that individuals being considered for BS are prone to exhibiting eating disorders and/or problematic eating behaviours, with research further indicating the potential persistence or emergence of these issues following the surgical procedure^11^. The diversity of eating-related behaviours and psychopathology presentations in BS patients have created inconsistencies in the literature in relation to the assessment methodology, diagnosis and treatment^12^.

These discrepancies could arise from factors like the duration of the follow-up period and differences in the utilization of assessment tools. Thus, presurgical psychological assessment should include instruments to evaluate psychopathological symptoms thoroughly. In that sense, enhancing comprehension of the psychological attributes exhibited by individuals undergoing BS would hold the potential to empower clinicians in pinpointing and effectively addressing distinct areas of concern^13^. Furthermore, such an understanding could potentially elucidate the extent to which motivational stages can serve as predictive indicators for behavioural changes^14^. Given that severe and inadequately managed mental health conditions, such as major depression, anxiety, bipolar disorder, and eating disorders, could potentially be viewed as contraindications for BS^15–17^, presurgical psychological assessment is usually focused on detecting severe mental disorders. However, mild intensity psychopathology may also influence postsurgical outcomes; therefore, multidimensional instruments capable of detecting mild intensity psychopathology and maladaptive eating behaviours in candidates for BS might enable treatment that might improve postsurgical outcomes.

An increasing number of assessment instruments have been validated for use in BS samples. The Personality Assessment Inventory (PAI) appears particularly useful for assessing candidates for BS^18^. Specifically, in this population, significantly high scores on the Positive Impression Management, Somatization, Depression, Dominance, and Warmth scales of the PAI are relevant^7,18^. A recent retrospective study found that psychological factors, as assessed by the PAI, affected long-term maintenance of weight loss after BS; anxiety and mania were associated with greater initial weight loss and faster weight regain after about 3 years, and alcohol problems were associated with worse BMI outcome^19^.

Moreover, binge eating stands out as the disorder most significantly impacted by BS. According to a comprehensive review, the prevalence of presurgical binge eating disorder spans from 2 to 49%, while subclinical binge eating behaviours are reported at rates ranging from 6 to 64% across various studies^20^. Notably, research indicates that the scores on the Binge Eating Scale (BES) show marked improvement postsurgery. Noteworthy findings also highlight a drastic reduction in the proportion of participants classified under the severe BES category, plummeting from 78% to a mere 5% (*p* < 0.01)^21^. Additionally, the severity of problematic eating behaviours, as assessed by the Eating Disorder Examination-Self-Report Questionnaire (EDE‐Q), demonstrates a consistent decline after surgery, maintaining levels lower than baseline throughout the follow-up period^22^. Given its validity within the Hispanic population, the EDE‐Q can be effectively integrated into preoperative psychological assessment batteries to screen for eating disorder psychopathology^23^. In light of these findings, both the binge eating scale (BES)^24^ and the Eating Disorder Examination-Self-Report Questionnaire (EDE‐Q)^25^ have proven to be robust instruments for the evaluation of these complex disorders.

In this study, we employed the PAI, EDE-Q, and BES to assess both the psychopathological profile and eating-related behaviours among individuals seeking bariatric surgery (BS), while also investigating potential changes in this profile 1-year post-surgery. The proposed mechanisms underlying the psychopathological shifts following BS are intertwined with the notion that individuals with obesity often contend with an adverse body image, thereby giving rise to responsive anxious-depressive manifestations and maladaptive conduct. BS, yielding substantial weight reduction, holds the potential to engender an amelioration in body image perception, subsequently culminating in the alleviation of maladaptive symptoms.

## Materials and methods

### Subjects

The recruitment of participants was on the basis of adding consecutive patients ready for BS during a period of time from March 2016 to March 2017 in a university hospital. Patients were considered ready after the selection by a multidisciplinary team led by an Endocrinologist and assisted by a Psychologist/Psychiatrist. All were aged ≥ 18 years with moderate-to-severe obesity, body mass index (BMI) ≥ 40 kg/m^2^ (or BMI ≥ 35 kg/m^2^, in patients with weight-related comorbidities), at least 6 months of failure to achieve and maintain weight loss in several diet programs, capacity to understand the surgical procedure and its effects, motivation for dietary monitoring (loss of 5% of weight in the 3 months prior to referral to surgery) and informed written consent to participate in the study. Exclusion criteria were: uncontrolled major psychiatric pathology or that prevents postoperative compliance, impaired judgment and/or active toxic habits, severe active eating disorder, unacceptable anesthetic risk, pregnancy or short-term desire, lactation, inflammatory bowel disease and evolutionary diseases of limited vital prognosis (neoplastic or systemic). All procedures involving the participants were performed according to the principles outlined in the Declaration of Helsinki, and the protocol was approved by the local ethics committee (CEIm 2015621).

### Design

In this prospective study, a multidimensional assessment inventory called PAI was employed to comprehensively assess psychopathological symptomatology^26–28^, and specific inventories for eating behaviour disorders, namely BES and EDE-Q^24,25^, were used to evaluate changes before and after BS, in comparison with normative data from the general population.

### Procedure

After endocrinological evaluation and a first appointment with the surgeon, candidates were referred to a psychiatrist for routine psychological evaluation comprising (a) a psychosocial interview including questions about personal and educational background, weight and weight loss trajectory, medical history, and adherence to medical recommendations; (b) the Structured Clinical Interview for DSM^29^; and (c) the Bulimic Investigatory Test, Edinburgh^30^. Patients were considered eligible for the study after this initial routine screening. Thereafter, they signed informed consent and were assessed with the PAI^26^, BES^24^ and EDE‐Q^25^. Patients were reassessed with these three instruments before BS and 1 year after surgery.

### Measures

The PAI^26^ is a self-administered test of personality and psychopathology features comprising 22 nonoverlapping full scales: 4 validity scales (infrequency, inconsistency, negative impression management, and positive impression management), 5 complementary validity scales (end questionnaire inconsistency, simulation index, Rogers discriminant function, defensiveness index, and Cashel discriminant function), 11 clinical scales (somatic complaints, anxiety, anxiety-related disorders, depression, mania, paranoia, schizophrenia, borderline features, antisocial features, alcohol problems, and drug problems), and 2 interpersonal scales (dominance and warmth). Ten of these scales are divided into 31 conceptually defined subscales. Participants qualify items on a Likert scale ranging from 1 (totally false, not at all true) to 4 (very true). The PAI also yields five treatment indicators (aggression, suicidal ideation, nonsupport, stress, and treatment rejection). To enable comparison with a census-matched community sample, scores on all PAI scales and subscales were transformed to T-scores. Transformed T-scores have a mean of 50 and a standard deviation of 10; thus, T-scores > 50 are above the mean value in the community sample, T-score > 60 is considered clinically relevant and T-scores ≥ 70 (two standard deviations above the mean) signify a marked deviation from the typical response in the community sample. Combining the clinical and personality scales and subscales can identify various psychopathological profiles, 15 clinical syndromes and 10 personality disorders^26,27^.

In addition, eating disorders were evaluated with these two questionnaires to detect mild altered behaviour pattern and attitudes in front of food. Firstly, the 36‐item EDE‐Q^25^ contains 14 attitudinal questions that assess the severity of eating pathology over the previous 28 days. Participants qualify items on a Likert scale ranging from 0 (none) to 6 (every day), with higher scores reflecting either greater severity or higher frequency of eating pathology. Item 1–15 and 29–36 generate the global score, which is the average score of four subscales: restraint, eating concern, weight concern, and shape concern. It is particularly valuable for assessing behaviour related to anorexia nervosa, bulimia nervosa, binge eating disorder, and other specified feeding and eating disorders. A score ≥ 5 points on the global scale and ≥ 5 on each of the subscales is considered clinically relevant.

The BES^24^ is a 16-item instrument widely used as a self-report measure of the severity and frequency of binge eating episodes and related psychological features. Participants respond to each item on a Likert scale, indicating the degree to which each statement applies to them. The scale assesses various aspects related to binge eating behaviour, including: frequency of binge eating, emotional aspects, loss of control, guilt and shame. The BES is useful for screening and diagnosing binge eating disorder, as well as tracking changes in binge eating behaviour over time, such as during treatment. A score of 17 is considered the most appropriate clinical cutoff point based on the sensitivity and specificity values of the test.

### Data analysis

Data from the PAI were prepared as recommended by Morey^26,27^: cases were excluded when (a) > 5% of questions were unanswered, (b) inconsistency T-scores were > 73, or (c) infrequency T-scores were > 75.

To test the normality of variables’ distribution, we used the Shapiro–Wilk test. In direct scores, we summarized normally distributed variables as means with standard deviations and non-normally distributed variables as medians and interquartile ranges. To compare with normative group, we used independent t-tests, the Mann–Whitney U, or chi-square tests, as appropriate. To compare pre-surgical and post-surgical parameters, we used paired t-tests, the Wilcoxon signed-rank test, or the McNemar test, as appropriate. Significance was set at *p* < 0.05. We utilized interclass correlation coefficients along with their corresponding 95% confidence intervals to assess the agreement or similarity between the test scores. We used Stata Statistical Software, version 13.1 (StataCorp LP; College Station, TX, USA) for all analyses.

### Ethical statement

All procedures involving the participants were performed according to the principles outlined in the Declaration of Helsinki, and the protocol was approved by the local ethics committee. This study protocol was reviewed and approved by the CEIm Committee, which is accredited by The Office For Human Research Protections of the United States Department of Health and Human Services, Federalwide Assurance (FWA), FWA00025624, approval number [CEIm 2015621].

### Consent to participate

Written informed consent was obtained from study participants. All of them signed a standard document to ensure the understanding of the objective and the signing the agreement to participate.

## Results

### Participants

A total of 42 individuals with moderate-to-severe obesity sought BS at our university hospital during this period of time. Of these, the routine screening excluded 6 for medical or surgical contraindications to BS and 4 for severe, active eating or mental disorders (1 individual met both exclusion criteria).

Thus, we analyzed data from 32 individuals: 21 (65.6%) women; mean age, 49.2 ± 6.8 years, range, 32‒59 years). At the presurgical evaluation, 22 (68.8%) were employed. BS consisted of sleeve gastrectomy in 15 (45.5%) patients and gastric bypass in 18 (54.5%). Twenty-six individuals were evaluated after surgery (loss to follow-up 18.75%) Mean BMI after surgery was 29.46 (5.1) kg/m^2^ after a mean follow-up of 14.1 (3.6) months.

Table 1 reports patients’ demographics and clinical history.

**Table 1:** Participants’ demographics and clinical history.

	Female (n = 21)	Male (n = 11)	Entire sample (n = 32)
Demographics			
Age in years, mean (sd)	49.2 (6.8)	44 (8.7)	47.5 (7.2)
Employed (%)	52.5	16.3	68.8
Presurgical clinical history			
Height (cm), mean (sd)	157.2 (7.3)	169.9 (11.3)	165.3 (15)
Weight (Kg), mean (sd)	118.7 (8.9)	138.2 (13.5)	127.1 (26.4)
BMI in kg/m^2^, mean (sd)	46.1 (6.1)	46.4 (7.8)	46.2 (6.6)
Body fat (%), mean (sd)	50.5 (4.3)	40.2 (5.1)	47.1 (4.7)
Hypertension, *n* (%)	14 (43.8)	5 (15.6)	59.4
Diabetes, *n* (%)	10 (31.3)	3 (9.4)	40.7
Dyslipidemia, *n* (%)	14 (43.8)	3 (9.4)	53.2
OSA *n* (%)	16 (50)	6 (18.8)	68.8
Osteoarticular pathology *n* (%)	1 (3.1)	1 (3.1)	6.2
Degenerative joint disease, *n* (%)	1 (3.1)	1 (3.1)	6.2
Eating disorder, *n* (%)	4 (12.5)	1 (3.1)	15.6
Depressive symptoms *n* (%)	6 (18.8)	1 (3.1)	21.9
Antihypertensive drugs *n* (%)	11 (34.4)	5 (15.6)	50
Hypolipidemic drugs *n* (%)	11 (34.4)	3 (9.4)	43.8
Antidiabetic drugs *n* (%)	9 (28.1)	2 (6.3)	34.4
Psychotropic drugs *n* (%)	11(34.4)	1 (3.1)	37.5

*sd* standard deviation, *BMI* body mass index, *OSA* obstructive sleep apnea.

### Comparison of PAI scales with the normative group before BS

The PAI was completed by 32 (100%) participants before surgery. None of the questionnaires were invalidated, because no T-scores on the validity scales were outside the acceptance protocol. Mean T-scores on the clinical scales ranged from 43.9 ± 9.7 for mania to 67.1 ± 14.1 for somatic concerns. The overall mean score 51.7 ± 10.5 was virtually identical to that of the standardization sample. The psychometric profile (stress 57 ± 13.2; anxiety 51 ± 11; depression 59.8 ± 13.3; somatic complaints 67 ± 14.1, and obsessive–compulsive disorder 49.7 ± 10.9) suggested that our cohort had mild somatic complaints with mild intensity mixed adjustment disorder. Table 2 shows the clinical scales, with the highest scores for somatic complaints and depression, however, only somatic complaints were high enough to be considered clinically relevant (T > 60). Table 3 shows the subscales’ scores. T-scores > 60 was considered clinically relevant. Among the somatic complaints, health concerns, conversion, and somatization together with the physiological depression subscale exhibited clinically relevant scores.

**Table 2:** Mean T-scores full-scale scores on the Personality Assessment Inventory before and after bariatric surgery.

PAI scale	Baseline (n = 32)	1 year after surgery (n = 26)	*P*-value
Validity scales			
Inconsistency	48.6 (10.1)	50.8 (12.7)	0.16
Frequency	50.2 (10.6)	50.9 (10.5)	0.24
Negative impression management	54.7 (13)	50.5 (8.4)	0.25
Positive impression management	52 (11.2)	50.7 (10.3)	0.01*
Clinical scales			
Somatic complaints	67.1 (14.1)	59.7 (15.6)	< 0.001*
Anxiety	50.8 (10.9)	48.7 (10.5)	0.64
Anxiety-related disorders	51.5 (11)	48.7 (9.3)	0.20
Depression	59.8 (13.3)	54.8 (10.5)	0.04*
Mania	43.9 (9.7)	44.6 (10.3)	0.18
Paranoia	50.7 (11.5)	48 (7.7)	0.33
Schizophrenia	50.5 (11.4)	47.8 (9.2)	0.53
Borderline features	49.5 (11.5)	47.5 (9.1)	0.96
Antisocial features	46.8 (9)	45.8 (8.1)	0.71
Alcohol problems	45.8 (7)	47.9 (11.7)	0.51
Drug problems	49.7 (9.2)	49.7 (9.6)	0.26
Treatment indicators			
Aggression	50.9 (9)	49.2 (8.2)	0.61
Suicidal ideation	52.7 (12.9)	49.6 (9.4)	0.59
Stress	57 (13.2)	52.7 (9.3)	0.65
Nonsupport	50.9 (12.1)	50.9 (8.2)	1.00
Treatment rejection	47.3 (11.6)	53.9 (11.3)	0.03*
Interpersonal scales			
Dominance	47.9 (9.4)	50.8 (9.8)	0.06
Warmth	51.8 (10.7)	53.1 (9.5)	0.37

*PAI* personality assessment inventory. T score > 60 is considered clinically relevant.

*p < 0.05, paired t-test or Wilcoxon test; results expressed as mean (standard deviation).

**Table 3:** Mean T-scores subscale scores on the Personality Assessment Inventory before and after bariatric surgery.

PAI subscale	Baseline (n = 32)	1 year after surgery (n = 26)	P-value
Somatic complaints			
Conversion	63.6 (14.8)	58.3 (17.2)	0.01*
Somatization	63.3 (11.9)	58.1 (13.7)	0.05*
Health concerns	66.9 (13.5)	58.6 (13.3)	< 0.001*
Anxiety			
Cognitive	49.6 (11)	48.2 (10.4)	0.83
Affective	49.4 (11.5)	47.3 (8.5)	0.77
Physiological	53 (10.2)	50.8 (13.5)	0.64
Anxiety-related disorders			
Obsessive–compulsive	49.7 (10.9)	45.7 (9.2)	0.04*
Phobias	50.9 (10.1)	50.8 (9)	0.92
Traumatic stress	52.2 (11.3)	48.1 (8.6)	0.08
Depression			
Cognitive	55.2 (13.1)	52 (9.4)	0.20
Affective	56.2 (14.8)	50.2 (11.8)	0.08
Physiological	62.6 (12.5)	58.8 (10.7)	0.06
Mania			
Activity level	46.2 (9.3)	50.8 (10)	< 0.001*
Grandiosity	45.9 (9.6)	44.9 (8.4)	0.75
Irritability	44.8 (9.8)	43 (9.5)	0.49
Paranoia			
Hypervigilance	47.7 (10.7)	45.5 (7.8)	0.35
Persecution	52.2 (12.3)	50 (6.9)	0.49
Resentment	52.1 (11.6)	49.6 (9.4)	0.56
Schizophrenia			
Psychotic experiences	49 (7.2)	46.4 (6.9)	0.19
Social detachment	50.1 (12.8)	50.2 (10.9)	0.49
Thought disorder	51.9 (11.7)	48 (9.9)	0.03*
Borderline features			
Affective instability	47.7 (10.2)	47 (7.8)	0.90
Identity problems	50.7 (13.8)	49 (10.5)	0.14
Negative relationships	49.9 (12)	47.8 (9.4)	0.29
Self-harm	49.7 (11.6)	49.3 (10.6)	0.54
Antisocial features			
Antisocial behaviors	48.3 (7.4)	46.7 (7.6)	0.78
Egocentricity	47.7 (14.7)	45.9 (7.1)	0.27
Stimulus seeking	45.9 (6.8)	47.7 (9.7)	0.30
Aggression			
Aggressive attitude	54 (8.5)	52.9 (10.1)	0.82
Verbal aggression	48.1 (8.5)	47.9 (8)	0.53
Physical aggression	50 (11.4)	46.8 (6.1)	0.11

*PAI* personality assessment inventory. T score > 60 is considered clinically relevant.

*p < 0.05, paired t-test or Wilcoxon test; results expressed as mean (standard deviation).

In summary, the presurgical psychometric profile suggested a mild intensity mixed adjustment disorder focused on somatic complaints. No psychopathological personality disorders were found. T-scores for other aspects in the psychometric profile were around 50, corresponding to the mean values in the general population.

### PAI full-scale and subscale profiles after BS

Twenty-six patients (81.25%) were assessed. At clinical scales, patients improved significantly after surgery on somatic complaints (SOM *p* < 0.001) and depression (DEP *p* = 0.04). At treatment-related scales, treatment rejection was also significantly increased (RTR *p* = 0.03) (Table 2).

In relation to subscales, after surgery, patients improved significantly in all somatic complaint areas (SOM-C *p* = 0.01; SOM-S *p* = 0.05; SOM-H *p* < 0.001), especially in concern for health (SOM-H: health concerns). Regarding anxiety-related disorders, the obsessive–compulsive symptoms decreased (TRA-O *p* = 0.04) and regarding psychotic disorders, cognitive distortions also decreased (ESQ-A *p* = 0.03) (Table 3).

### Eating behaviour before and after BS

Before surgery, the absence of eating behaviour disorders is noted in our cohort. In BES n = 32 (100%) the mean score was 5.29 ± 4.12 and in EDE-Q/G n = 32 (100%) the global men score was 2.44 ± 0.88. Restrictive eating behaviour and cognitive symptoms of concern for weight and body shape improved significantly after surgery, becoming in line with the values of the general population. After surgery N = 26 (81.25%), significant improvements were observed in scores for compulsive intake (BES *p* < 0.001), restrictive behaviour (EDE-Q/R *p* < 0.001), concern for weight (EDE-Q/W *p* < 0.001), shape concern (EDE-Q/S *p* < 0.001), and overall key behaviours of eating disorders and related cognitive symptoms (EDE-Q/G *p* < 0.001). After surgery, patients reported lower occurrence and frequency of key behavioural traits in eating disorders, including compulsive eating, misuse of laxatives and diuretics, and concern for weight and body shape. However, concern for food did not change significantly (EDE-Q/E *p* = 0.73) (Table 4).

**Table 4:** Eating behaviour data.

	Baseline (n = 32) mean (sd)	1 year after surgery (n = 26) mean (sd)	*P*-value
Binge eating scale	5.29 (4.12)	2.5 (2.47)	< 0.001*
EDE-Q/R: restraint	3.24 (1.10)	1.91 (1.21)	< 0.001*
EDE-Q/E: eating concern	0.52 (0.74)	0.49 (0.57)	0.73
EDE-Q/W: weight concern	2.73 (1.30)	1.26 (1.13)	< 0.001*
EDE-Q/S: shape concern	2.96 (1.56)	1.67 (1.48)	< 0.001*
EDE-Q/G: global score	2.44 (0.88)	1.34 (0.80)	< 0.001*

*sd* standard deviation, *EDE-Q* eating disorder examination self-report questionnaire. *BES*: a score of 17 is deemed the optimal clinical cutoff based on test sensitivity and specificity, *EDE-Q* a score of ≥ 5 on the global scale and each subscale is considered clinically relevant.

*p < 0.05, paired t-test or Wilcoxon test; results expressed as mean (standard deviation).

## Discussion

BS can be an effective treatment for obesity, although the presence of psychopathology, even of mild intensity or subclinical, eating-related behaviours and certain personality traits may affect postsurgical outcomes^19^. To our knowledge, this is the first study to prospectively assess patients with the PAI before and after BS. Our findings using the multidimensional PAI add strength and depth to those reported in previous studies using unidimensional psychological tests that reported a higher prevalence of mental health disorders (especially symptoms of depression or anxiety) in people with obesity as well as improvements after BS^31–36^.

In the present study by PAI, mild somatic complaints, especially health concerns, depressive and anxiety symptoms and compulsive personality traits were more common in participants than in the general population. We found that patients’ presurgical profile was compatible with a mild intensity mixed (anxious-depressive) adjustment disorder. In this sense, the only scale with mean clinically relevant scores (T > 60) was somatic complaints, particularly health concerns, characterized by excessive concern about having a serious illness or the likelihood of becoming ill despite a lack of medical evidence for illness. This dimension is closely related to depression and stress, which also involve feelings of worry and anxiety, while generating frustration and helplessness. A comparable psychopathological clinical profile has been previously documented utilizing alternative instruments to the PAI in 15% of obese patients awaiting BS or in hyperphagic obesity up to 30%^22^. In the same direction as the PAI, the baseline EDE-Q identified symptoms of cognitive anxiety about weight and body shape that were closely related to a somatic complaint, and these symptoms significantly improved 1 year after surgery. Moreover, while the PAI did not diagnose any personality disorders among our patients, it did illuminate certain associated personality traits. These traits were typified by a compulsive and obsessive behavioural pattern, alongside the presence of distorted or maladaptive thoughts concerning their cognitive perceptions of health status. According to the results of the PAI, after surgery, the profile improved by a noteworthy decrease in somatic complaints and anxious-depressive symptoms, enhanced adherence to therapeutic measures, reduced compulsive behaviour, and diminished cognitive distortion. Additionally, there was a substantial increase in patients’ activity. This profile was more linear and closer to that of the general population, although somatic complaints and affective disturbances remained more common in our cohort than in the general population.

Eating disorders were less prevalent and exhibited milder severity within our cohort compared to findings from other studies, as indicated by lower presurgical scores on the EDE-Q global scale^4,10,22^. This difference can likely be attributed to our standard presurgical evaluation process, which systematically identifies and excludes severe eating disorders that could potentially compromise surgical outcomes^37–39^. We found no symptoms of compulsive intake or diagnostic criteria for binge eating disorder in BES, although restrictive eating behaviours and cognitive symptoms of concern for weight and body shape were common. Compulsive and obsessive behaviour and eating disturbances both entail easy frustration at having to wait for rewards; action in individuals with these traits is often geared toward achieving immediate satisfaction^40^ and avoiding situations that do not provide immediate reinforcement or that require perseverance to achieve rewards.

Following BS, notable enhancements were observed in various facets of psychopathology, particularly in somatic complaints, the anxious-depressive syndrome, and cognitive distortions. Additionally, there was a marked amelioration in restrictive eating behaviours and disturbances related to body image subsequent to BS. A previous study already reported an improvement in the BES score after surgery and a decline of the percentage of patients in the severe category from 78 to 5% (*p* < 0.01)^21^. Similarly, in our cohort, mean BES score was reduced in 2.79 points after surgery, although the score was in the category of not clinically relevant across the duration of the study. According with others^22^ the severity of problematic eating behaviours measured by EDE‐Q decreased up to 54.9% after surgery and remained lower than baseline throughout follow-up. Importantly, a majority of these symptoms (including compulsive eating, misuse of laxatives and diuretics, concern for weight and body shape) also exhibited improvement post-surgery, with patients’ post-surgical scores aligning closely with those observed in the general population. These findings underscore the potential for BS to effectively mitigate mild eating disorders, as well as alleviate mild psychopathological symptoms and maladaptive thought patterns.

Other findings^41^ support the notion that a significant portion of the improvements in mental health can be attributed to the weight loss itself, resulting in positive changes in body image, self-esteem, and self-concept. Nevertheless, it is important to acknowledge that other factors also contribute to postoperative mental well-being, including the patient’s perception of regained control over their life and the support received from healthcare professionals. Therefore, substantial weight loss together with the perception of the own personal progress and recovering the command over one’s life has the potential to foster an enhanced perception of body image, subsequently leading to a reduction in maladaptive symptoms, particularly in cases of mild mixed adjustment disorder.

Numerous studies have examined psychopathology, personality traits, and eating disorders within the bariatric population; however, few have delved into the intricate interconnections among these psychological, personality, and eating aspects among individuals with obesity who are pursuing BS^42^. The utilization of the PAI offers a comprehensive tool to illuminate both psychopathological tendencies and personality disorders, thereby pinpointing the pivotal psychological attributes that define these individuals. Furthermore, it aids in evaluating these attributes as potential predictors of surgical outcomes. Consequently, these psychological and personality variables warrant consideration for inclusion in psychotherapeutic interventions aimed at mitigating psychological distress and maladaptive eating behaviours. These findings underscore the significance of incorporating personality and psychological variables into the pre-surgery assessment process, as well as the potential utility of targeting them for psychotherapeutic interventions either prior to or following the surgical procedure.

Several limitations inherent to our study warrant consideration. Primarily, the modest sample size has likely curtailed the statistical robustness of our analyses, potentially compromising the sensitivity of certain investigations. Furthermore, the limited sample size impeded the execution of a comprehensive prognostic analysis and the incorporation of adjustments for bias and confounding variables. It is noteworthy, however, that a mere 18.75% of patients were lost to follow-up, a retention rate that proves favorable when contrasted with analogous obesity-focused investigations. Secondly, this longitudinal exploratory inquiry abstained from contrasting subjects with a control group (only with normative data), constituting a prospective analysis restricted to a solitary year of follow-up and bereft of supplementary data. As a result, any predictions drawn from the study’s findings must be approached with caution. It is essential for future investigations to encompass control groups and larger cohorts observed over an extended period of follow-up.

In conclusion, in our cohort, candidates for BS have somatic complaints (especially concern for their health), mild affective disorder (depression) and stress, restrictive eating behaviours, and cognitive symptoms of concern for weight and body shape, but they do not have a formal eating or personality disorder. Most of these symptoms improve after BS. Further studies are needed to evaluate whether improvements in mild intensity psychopathology benefits outcomes after BS.

## Data Availability

All data generated or analyzed during this study are included in this article. Further enquiries can be directed to the corresponding author.

## References

[1] Oltmanns JR, Rivera Rivera J, Cole J, Merchant A, Steiner JP (2020). Personality psychopathology: Longitudinal prediction of change in body mass index and weight post-bariatric surgery. Health Psychol..

[2] Sarwer DB, Cohn NI, Gibbons LM, Magee L, Crerand CE, Raper SE (2004). Psychiatric diagnoses and psychiatric treatment among bariatric surgery candidates. Obes Surg..

[3] Caixàs A, Lecube A, Morales MJ, Calañas A, Moreiro J, Cordido F (2013). Group for the study of obesity of the Spanish Endocrinology and nutrition society (SEEN), Crosby RD, Kolotkin RL. Weight-related quality of life in Spanish obese subjects suitable for bariatric surgery is lower than in their North American counterparts: a case-control study. Obes Surg..

[4] Peterhänsel C, Linde K, Wagner B, Dietrich A, Kersting A (2017). Subtypes of personality and ‘locus of control’ in bariatric patients and their effect on weight loss, eating disorder and depressive symptoms, and quality of life. Eur. Eat. Disord. Rev..

[5] García-Ruiz-de-Gordejuela A, Agüera Z, Granero R, Steward T, Llerda-Barberá A, López-Segura E (2017). Weight loss trajectories in bariatric surgery patients and psychopathological correlates. Eur. Eat. Disord. Rev..

[6] de Zwaan M, Enderle J, Wagner S, Mühlhans B, Ditzen B, Gefeller O (2011). Anxiety and depression in bariatric surgery patients: a prospective, follow-up study using structured clinical interviews. J. Affect. Disord..

[7] Walter FA, Hoyt T, Martinez H, Dziura J (2022). Preoperative psychological assessment and weight loss outcomes in bariatric surgery patients at a military treatment facility: A retrospective profile analysis. Mil Med..

[8] Livhits M, Mercado C, Yermilov I, Parikh JA, Dutson E, Mehran A (2012). Preoperative predictors of weight loss following bariatric surgery: systematic review. Obes Surg..

[9] Courcoulas AP, Christian NJ, O’Rourke RW, Dakin G, Patchen Dellinger E, Flum DR, Melissa Kalarchian PD, Mitchell JE, Patterson E, Pomp A, Pories WJ, Spaniolas K, Steffen K, Wolfe BM, Belle SH (2015). Preoperative factors and 3-year weight change in the Longitudinal Assessment of Bariatric Surgery (LABS) consortium. Surg. Obes. Relat. Dis..

[10] Gero D, Tzafos S, Milos G, Gerber PA, Vetter D, Bueter M (2019). Predictors of a healthy eating disorder examination-questionnaire (EDE-Q) score 1 year after bariatric surgery. Obes. Surg..

[11] Gordon EL, Terrill AL, Smith TW, Ibele AR, Martinez P, McGarrity LA (2022). Overvaluation of shape and weight (Not BMI) associated with depressive symptoms and binge eating symptoms Pre- and Post-bariatric surgery. Obes. Surg..

[12] Conceição EM, Utzinger LM, Pisetsky EM (2015). Eating disorders and problematic eating behaviours before and after bariatric surgery: Characterization, assessment and association with treatment outcomes. Eur. Eat. Disord. Rev..

[13] Hood MM, Corsica JA, Azarbad L (2011). Do patients seeking laparoscopic adjustable gastric banding surgery differ from those seeking gastric bypass surgery? A comparison of psychological profiles across ethnic groups. Obes. Surg..

[14] Lecube A, Sánchez E, Andrés A, Saldaña C, Morales MJ, Calañas A (2019). Obesity group of the spanish society of endocrinology and nutrition (GOSEEN). Assessing motivational stages and processes of change for weight management around bariatric surgery: A multicenter study. Obes. Surg..

[15] Pull CB (2010). Current psychological assessment practices in obesity surgery programs: What to assess and why. Curr. Opin. Psychiatry.

[16] Neovius M, Bruze G, Jacobson P, Sjöholm K, Johansson K, Granath F (2018). Risk of suicide and non-fatal self-harm after bariatric surgery: Results from two matched cohort studies. Lancet Diabetes Endocrinol..

[17] Mitchell JE, de Zwaan M (2011). Psychosocial assessment and treatment of bariatric surgery patients.

[18] Corsica JA, Azarbad L, McGill K, Wool L, Hood M (2010). The personality assessment inventory: Clinical utility, psychometric properties, and normative data for bariatric surgery candidates. Obes. Surg..

[19] Hoyt T, Walter FA (2022). The relationship of presurgical personality assessment Inventory scales to BMI following bariatric surgery. Health Psychol..

[20] Niego SH, Kofman MD, Weiss JJ, Geliebter A (2007). Binge eating in the bariatric surgery population: A review of the literature. Int. J. Eat. Disord..

[21] Jabbour G, Salman A (2021). Bariatric surgery in adults with obesity: the impact on performance, metabolism, and health indices. Obes. Surg..

[22] Nasirzadeh Y, Kantarovich K, Wnuk S, Okrainec A, Cassin SE, Hawa R, Sockalingam S (2018). Binge eating, loss of control over eating, emotional eating, and night eating after bariatric surgery: results from the Toronto Bari-PSYCH Cohort Study. Obes Surg..

[23] Marek RJ, Ivezaj V, Parikh MS, Jayade M, Davila-Shiau E, Grilo CM (2023). Factor structure and measurement invariance of the English-versus Spanish-language eating disorder examination questionnaire: brief form (S-EDE-Q-BF) in Hispanic/Latino/a/x persons seeking bariatric surgery. Surg. Obes. Relat. Dis..

[24] Zuñiga O, Robles I, Ramírez A, Páez F (2006). Evaluación del trastorno por atracón en población mexicana: traducción y propiedades psicométricas de la versión en español del cuestionario de trastorno por atracón. Psiquiatría.

[25] Peláez-Fernández MA, Labrador FJ, Raich RM (2012). Validation of eating disorder examination questionnaire (EDE-Q)—Spanish Version for screening eating disorders. Span. J. Psychol..

[26] Ortiz-Tallo M, Cardenal V, Ferragut M, Santamaría P (2015). Spanish and chilean standardizations of the personality assessment inventory: the influence of sex. Span J. Psychol..

[27] Ortiz-Tallo M, Cardenal V, Sánchez MP (2012). Guía de interpretación y evaluación de casos clínicos con el Inventario de Evaluación de la Personalidad (PAI).

[28] Burneo-Garcés C, Fernández-Alcántara M, Aguayo-Estremera R, Pérez-García M (2020). Psychometric properties of the spanish adaptation of the personality assessment inventory in correctional settings: An ESEM study. J. Pers. Assess..

[29] First MB, Spitzer RL, Gibbon M, Williams JBW (2002). Structured clinical interview for DSM-IV-TR Axis I disorders (SCID-I).

[30] Vaz Leal FJ, Peñas Lledó EM (1999). Estudio diferencial de las formas completas y subclínicas de bulimia nerviosa [Differential study of the complete and subclinical presentations of bulimia nervosa]. Actas. Esp. Psiquiatr..

[31] Kalarchian MA, King WC, Devlin MJ, Hinerman A, Marcus MD, Yanovski SZ (2019). Mental disorders and weight change in a prospective study of bariatric surgery patients: 7 years of follow-up. Surg. Obes. Relat. Dis..

[32] Duarte-Guerra LS, Coêlho BM, Santo MA, Wang YP (2015). Psychiatric disorders among obese patients seeking bariatric surgery: Results of structured clinical interviews. Obes. Surg..

[33] Kinzl JF, Schrattenecker M, Traweger C, Mattesich M, Fiala M, Biebl W (2006). Psychosocial predictors of weight loss after bariatric surgery. Obes. Surg..

[34] van Hout G, van Heck G (2009). Bariatric psychology, psychological aspects of weight loss surgery. Obes. Facts..

[35] Lin HY, Huang CK, Tai CM, Lin HY, Kao YH, Tsai CC (2013). Psychiatric disorders of patients seeking obesity treatment. BMC Psychiatry.

[36] León RT, Zumaeta VA, Ruiz PS (2017). The complex relationship between mental health and weight loss surgery. A review. Rev. Chil. Cir..

[37] Smith CE, Hawkins MAW, Williams-Kerver GA, Duncan J (2020). Depression subtypes, binge eating, and weight loss in bariatric surgery candidates. Surg. Obes. Relat. Dis..

[38] Smith KE, Orcutt M, Steffen KJ, Crosby RD, Cao L, Garcia L (2019). Loss of control eating and binge eating in the 7 years following bariatric surgery. Obes. Surg..

[39] Sarwer DB, Allison KC, Wadden TA, Ashare R, Spitzer JC, McCuen-Wurst C (2019). Psychopathology, disordered eating, and impulsivity as predictors of outcomes of bariatric surgery. Surg. Obes. Relat. Dis..

[40] Pujol J, Blanco-Hinojo L, Martínez-Vilavella G, Deus J, Pérez-Sola V, Sunyer J (2021). Dysfunctional brain reward system in child obesity. Cereb Cortex..

[41] Kubik JF, Gill RS, Laffin M, Karmali S (2013). The impact of bariatric surgery on psychological health. J. Obes..

[42] Monteleone AM, Cascino G, Solmi M, Pirozzi R, Tolone S, Terracciano G, Parisi S, Cimino M, Monteleone P, Maj M, Docimo L (2019). A network analysis of psychological, personality and eating characteristics of people seeking bariatric surgery: Identification of key variables and their prognostic value. J. Psychosom. Res..

